# Population Dynamics of Escherichia coli Causing Bloodstream Infections over Extended Time Periods

**DOI:** 10.1128/msphere.00956-21

**Published:** 2021-12-22

**Authors:** Johann D. D. Pitout

**Affiliations:** a Alberta Precision Laboratories, Calgary, Alberta, Canada; b Cummings School of Medicine, University of Calgary, Calgary, Alberta, Canada; c University of Pretoria, Pretoria, Gauteng, South Africa

**Keywords:** *Escherichia coli*, ST131, bloodstream infections

## Abstract

Escherichia coli is a leading cause of community-acquired and health care-associated bloodstream infections (BSIs) worldwide. Limited information is available regarding the changes in population dynamics of human E. coli over extended time periods, especially among nonbiased E. coli isolates in large well-defined geographical regions. Coque and colleagues (I. Rodríguez, A. S. Figueiredo, M. Sousa, S. Aracil-Gisbert, et al., mSphere 6:e00868-21, 2021, https://doi.org/10.1128/msphere.00868-21) conducted a longitudinal study of E. coli BSIs in a Madrid hospital over a 21-year period (1996 to 2016). Certain E. coli B2 phylogroups (i.e., ST131 and ST73) dominated the community E. coli population in Madrid. These community clones were often introduced into the hospital setting. This study and other longitudinal surveys from England and Canada showed that ST131 subclades C1 and C2 were mainly responsible for the increase in fluoroquinolone and cephalosporin resistance among E. coli during the mid- to late 2000s.

## COMMENTARY

Escherichia coli is the most common cause of community-acquired and health care-associated bloodstream infections (BSIs) worldwide and is responsible for substantial health care costs and patient morbidity and mortality (1). E. coli BSIs are associated with increased in-hospital deaths, and resistance to front-line antibiotics prolonged the length of hospital stay (2).

In the 1990s, E. coli was susceptible to most antibiotic classes (3). During the 2000s, multidrug resistance (MDR) increased exponentially, especially to fluoroquinolones and third-generation cephalosporins (4). These drugs are often used to treat serious E. coli infections (5). This led to overuse of certain antibiotics such as carbapenems and increased carbapenem resistance. Losing the use of the carbapenems will be devastating for medical practice. They are the most effective last-line treatment options for MDR E. coli serious infections (6).

The population structure of human E. coli is dominated by the following sequence types (STs) (7): ST131, ST69, ST10, ST405, ST38, ST95, ST648, ST73, ST410, ST393, ST354, ST12, ST127, ST167, ST58, ST617, ST88, ST23, ST117, and ST1193. The prevalence of these STs depends on geographic location, inclusion criteria, sources, and time periods of studies (7).

ST131 is the most widespread clone among human E. coli isolates. It causes millions of human infections and deaths annually (8). ST131 belongs to three clades: susceptible A, B, and fluoroquinolone-/cephalosporin-resistant C. ST131-B acquired fluoroquinolone resistance to become ST131-C (9). ST131-C then split into subclades (C0, C1, C1_M27, and C2) which became cephalosporin resistant by acquiring different CTX-M plasmids (*bla*_CTX-M-14_ with C1, *bla*_CTX-M-27_ with C1_M27, and *bla*_CTX-M-15_ with C2).

Limited information is available regarding the changes in population structure of human E. coli isolates over extended time periods, especially among nonbiased E. coli isolates in large well-defined geographical regions. Such information will aid with designing superior treatment and prevention strategies for E. coli BSIs.

To address this issue, Coque and colleagues conducted a longitudinal study of E. coli BSIs in a Madrid hospital over a 21-year period (1996 to 2016) (10). They used multiplex PCR and pulsed-field gel electrophoresis (PFGE) to classify 649 (representing 10% of the total population) E. coli isolates into major phylogroups (A, B1, B2 subgroups, C, D, E, and F). Isolates (*n* = 45) representing highly related pulsotypes also underwent short-read whole-genome sequencing (WGS).

The E. coli BSI incidence increased from 5.5 (1996) to 10.8 (2016) BSI episodes/1,000 hospital admissions. E. coli phylogroup B2 was common and mainly obtained from community patients. Other E. coli phylogroups (D, B1, A, C, F, and E) were rare.

STc131 (phylogroup B2-I) was the most common and MDR clone in the Madrid study (i.e., 12.3% of the E. coli population). This ST often caused infections in the elderly (>80 years). The fluoroquinolone-resistant ST131 C subclades (C1 and C2) were dominant (71%), while ST131-A (9%) and ST131-B (20%) were rare. ST131-B was present among E. coli isolates causing BSIs during 1996. ST131-C0 was first detected in 2000, while ST131-C1 and ST131-A appeared during 2004. This was followed by ST131-C2 (with *bla*_CTX-M-15_) in 2006 and ST131-C1_M27 (with *bla*_CTX-M-27_) in 2016. These interesting longitudinal data support the current understanding of ST131 evolution and expansion (11). Subclades ST131-C1 and -C2 were mainly responsible for the increase in fluoroquinolone and cephalosporin resistance during the mid- to late 2000s. The authors also identified the following dominant phylogroup B2 clones: STc73 (B2-II), STc127 (B2-III), STc-95 (B2-IX), and STc12 (B2-VI). The incidences of these other epidemic phylogenetic B2 groups varied throughout the study period. As with STc131, they persisted in the Madrid E. coli population for long time periods (up to 17 years).

Coque and colleagues showed that the increased incidence of hospital E. coli BSIs was tied to community epidemics (10). Their data indicated that certain E. coli B2 phylogroups (i.e., STc131 and STc73) dominated the Madrid community E. coli population and that these clones are often introduced into the hospital setting. Community “outbreaks” often predated the hospital dissemination. Such clones were seldom the cause of large hospital-acquired outbreaks.

Comparable E. coli BSI longitudinal studies were undertaken in England and Calgary, Canada. The England study undertook an 11-year (2001 to 2011) longitudinal hospital-based survey (12). They included 1,509 blood E. coli isolates obtained from 11 hospitals across England. Overall, the most common STs were ST73 (17%), ST131 (14%), ST95 (11%), and ST69 (6%). ST131 was first detected in 2003. The ST131 population was also dominated by MDR C subclades. The prevalence of ST131 clades remained stable from 2006 to 2011. Therefore, the expansion of ST131 was not driven by sequential introduction of increasingly resistant subclades (12). The proportion of other common STs (i.e., ST73, ST95, and ST69) remained unchanged over the study period.

The Calgary population-based surveillance is different from those in Madrid and England in that surveys (1999 to 2016) included all community and hospital E. coli isolates causing BSIs in a region of 1.4 million people (13). The most common STs were ST131 (21% of 686 isolates), ST73 (14%), ST69 (8%), ST95 (7%), and ST1193 (5%). ST131 infected mostly the elderly in long-term-care centers (14) (especially ST131-C1 with *bla*_CTX-M-14_ and ST131-C1_M27 with *bla*_CTX-M-27_). ST131 was relatively rare among E. coli BSIs during the early 2000s but increased significantly toward the latter part of the 2000s (15, 16). The overall prevalence of ST131 among total E. coli isolates from blood increased from 53/481 (11%) in 2006 to 150/621 (24.2%) in 2012 and 141/684 (20.6%) in 2016 (17). ST131-C2 was first detected in 1999, and ST131-C1 in 2001 and ST131-C1_M27 in 2006 (16). As with the Madrid study, MDR C clades dominated the ST131 population. However, the proportion of the ST131 clades changed over time, which is different from the England and Madrid studies. ST131-C1 and ST131-B were common in 2006, while ST131-C2 with *bla*_CTX-M-15_, C1-M27 with *bla*_CTX-M-27_, and ST131-A significantly increased from 2012 onward. ST131-C2 was the most MDR subclade in Calgary and increased exponentially over time (17).

ST1193 is an emerging global fluoroquinolone-resistant clone, especially in the United States (18, 19). In Calgary, ST1193 is increasingly significant among E. coli BSIs (2016 to 2018), especially among long-term-care residents (20). The rapid emergence of ST1193 is concerning and is adding to the public health burden of global MDR E. coli BSIs.

The Madrid study provided fascinating information regarding longitudinal population dynamics of E. coli STs (especially ST131) causing BSIs. Eliminating ST131 (especially the MDR C subclades) would substantially decrease the overall global incidence rate and MDR burden within E. coli isolates causing hospital BSIs. This will lead to considerable worldwide public health benefits.

## References

[1] Laupland KB, Church DL. 2014. Population-based epidemiology and microbiology of community-onset bloodstream infections. Clin Microbiol Rev 27:647–664. doi:10.1128/CMR.00002-14. 25278570 PMC4187633

[2] Naylor NR, Pouwels KB, Hope R, Green N, Henderson KL, Knight GM, Atun R, Robotham JV, Deeny SR. 2019. The health and cost burden of antibiotic resistant and susceptible Escherichia coli bacteraemia in the English hospital setting: a national retrospective cohort study. PLoS One 14:e0221944. doi:10.1371/journal.pone.0221944. 31504046 PMC6736296

[3] Pitout JD. 2012. Extraintestinal pathogenic Escherichia coli: an update on antimicrobial resistance, laboratory diagnosis and treatment. Expert Rev Anti Infect Ther 10:1165–1176. doi:10.1586/eri.12.110. 23199402

[4] Pitout JD, Chan WW, Church DL. 2016. Tackling antimicrobial resistance in lower urinary tract infections: treatment options. Expert Rev Anti Infect Ther 14:621–632. doi:10.1080/14787210.2016.1188004. 27177113

[5] Peirano G, Pitout JDD. 2019. Extended-spectrum beta-lactamase-producing Enterobacteriaceae: update on molecular epidemiology and treatment options. Drugs 79:1529–1541. doi:10.1007/s40265-019-01180-3. 31407238

[6] Papp-Wallace KM, Endimiani A, Taracila MA, Bonomo RA. 2011. Carbapenems: past, present, and future. Antimicrob Agents Chemother 55:4943–4960. doi:10.1128/AAC.00296-11. 21859938 PMC3195018

[7] Manges AR, Geum HM, Guo A, Edens TJ, Fibke CD, Pitout JDD. 2019. Global extraintestinal pathogenic *Escherichia coli* (ExPEC) lineages. Clin Microbiol Rev 32:e00135-18. doi:10.1128/CMR.00135-18. 31189557 PMC6589867

[8] Pitout JD, DeVinney R. 2017. Escherichia coli ST131: a multidrug-resistant clone primed for global domination. F1000Res 6:195. doi:10.12688/f1000research.10609.1. PMC5333602 28344773

[9] Mathers AJ, Peirano G, Pitout JD. 2015. The role of epidemic resistance plasmids and international high-risk clones in the spread of multidrug-resistant *Enterobacteriaceae*. Clin Microbiol Rev 28:565–591. doi:10.1128/CMR.00116-14. 25926236 PMC4405625

[10] Rodríguez I, Figueiredo AS, Sousa M, Aracil-Gisbert S, Fernández de Bobadilla MD, Lanza VF, Rodríguez C, Zamora J, Loza E, Mingo P, Brooks CJ, Cantón R, Baquero F, Coque TM. 2021. A 21-year survey of *Escherichia coli* from bloodstream infections (BSI) in a tertiary hospital reveals how community-hospital dynamics of B2 phylogroup clones influence local BSI rates. mSphere 6:e00868-21. doi:10.1128/msphere.00868-21. PMC8722714 34935444

[11] Pitout JDD, Finn TJ. 2020. The evolutionary puzzle of Escherichia coli ST131. Infect Genet Evol 81:104265. doi:10.1016/j.meegid.2020.104265. 32112974

[12] Kallonen T, Brodrick HJ, Harris SR, Corander J, Brown NM, Martin V, Peacock SJ, Parkhill J. 2017. Systematic longitudinal survey of invasive Escherichia coli in England demonstrates a stable population structure only transiently disturbed by the emergence of ST131. Genome Res 27:1437–1449. doi:10.1101/gr.216606.116. PMC5538559 28720578

[13] Holland MS, Nobrega D, Peirano G, Naugler C, Church DL, Pitout JDD. 2020. Molecular epidemiology of Escherichia coli causing bloodstream infections in a centralized Canadian region: a population-based surveillance study. Clin Microbiol Infect 26:1554.e1–1554.e8. doi:10.1016/j.cmi.2020.02.019. 32120035

[14] Nobrega D, Peirano G, Lynch T, Finn TJ, Devinney R, Pitout JDD. 2021. Spatial distribution of Escherichia coli ST131 C subclades in a centralized Canadian urban region. J Antimicrob Chemother 76:1135–1139. doi:10.1093/jac/dkab020. 33547472

[15] Peirano G, Pitout JD. 2014. Fluoroquinolone-resistant *Escherichia coli* sequence type 131 isolates causing bloodstream infections in a Canadian region with a centralized laboratory system: rapid emergence of the H30-Rx sublineage. Antimicrob Agents Chemother 58:2699–2703. doi:10.1128/AAC.00119-14. 24566175 PMC3993220

[16] Peirano G, van der Bij AK, Gregson DB, Pitout JD. 2012. Molecular epidemiology over an 11-year period (2000 to 2010) of extended-spectrum beta-lactamase-producing *Escherichia coli* causing bacteremia in a centralized Canadian region. J Clin Microbiol 50:294–299. doi:10.1128/JCM.06025-11. 22162555 PMC3264152

[17] Peirano G, Lynch T, Matsumara Y, Nobrega D, Finn TJ, DeVinney R, Pitout JDD. 2020. Trends in population dynamics of Escherichia coli sequence type 131, Calgary, Alberta, Canada, 2006–2016. Emerg Infect Dis 26:2907–2915. doi:10.3201/eid2612.201221. 33219650 PMC7706940

[18] Johnson TJ, Elnekave E, Miller EA, Munoz-Aguayo J, Flores Figueroa C, Johnston B, Nielson DW, Logue CM, Johnson JR. 2019. Phylogenomic analysis of extraintestinal pathogenic *Escherichia coli* sequence type 1193, an emerging multidrug-resistant clonal group. Antimicrob Agents Chemother 63:e01913-18. doi:10.1128/AAC.01913-18. 30348668 PMC6325179

[19] Tchesnokova VL, Rechkina E, Larson L, Ferrier K, Weaver JL, Schroeder DW, She R, Butler-Wu SM, Aguero-Rosenfeld ME, Zerr D, Fang FC, Ralston J, Riddell K, Scholes D, Weissman S, Parker K, Spellberg B, Johnson JR, Sokurenko EV. 2019. Rapid and extensive expansion in the United States of a new multidrug-resistant Escherichia coli clonal group, sequence type 1193. Clin Infect Dis 68:334–337. doi:10.1093/cid/ciy525. 29961843 PMC6321845

[20] Peirano G, Matsumara Y, Nobrega D, DeVinney R, Pitout J. 2021. Population-based epidemiology of Escherichia coli ST1193 causing blood stream infections in a centralized Canadian region. Eur J Clin Microbiol Infect Dis doi:10.1007/s10096-021-04373-5. 34750697

